# Primary Care Providers’ Level of Preparedness for Recommending Physical Activity to Adults With Disabilities

**DOI:** 10.5888/pcd14.170328

**Published:** 2017-11-16

**Authors:** Elizabeth A. Courtney-Long, Alissa C. Stevens, Dianna D. Carroll, Shannon Griffin-Blake, John D. Omura, Susan A. Carlson

**Affiliations:** 1 Division of Human Development and Disability, National Center on Birth Defects and Developmental Disabilities, Centers for Disease Control and Prevention, Atlanta, Georgia; 2 Commissioned Corps, US Public Health Service, Atlanta, Georgia; 3 Division of Nutrition, Physical Activity, and Obesity, National Center for Chronic Disease Prevention and Health Promotion, Centers for Disease Control and Prevention, Atlanta, Georgia

## Abstract

**Introduction:**

Adults with disabilities are more likely to be physically inactive than those without disabilities. Although receiving a health care provider recommendation is associated with physical activity participation in this population, there is little information on factors associated with primary care providers recommending physical activity to patients with disabilities.

**Methods:**

We used 2014 DocStyles data to assess primary care provider characteristics and perceived barriers to and knowledge-related factors of recommending physical activity to adult patients with disabilities, by how prepared primary care providers felt in making recommendations. We used log-binomial regression to estimate adjusted prevalence ratios (PRs) and 95% confidence intervals (CIs) between recommending physical activity at most visits and primary care provider characteristics and preparedness.

**Results:**

Most primary care providers strongly (36.3%) or somewhat (43.3%) agreed they felt prepared to recommend physical activity to patients with disabilities. We found significant trends between preparedness and primary care provider age (*P* = .001) and number of patients with disabilities seen per week (*P* < .001). Half (50.6%) of primary care providers recommend physical activity to patients with disabilities at most visits. Primary care providers who strongly agreed (adjusted PR, 1.74; 95% CI, 1.44–2.09) or somewhat agreed (adjusted PR, 1.36; 95% CI, 1.22–1.65) they felt prepared were more likely to recommend physical activity at most visits compared with those who were neutral or disagreed.

**Conclusion:**

Primary care providers are more likely to recommend physical activity to patients with disabilities regularly if they feel prepared. Understanding factors and barriers associated with preparedness can help public health programs develop and disseminate resources for primary care providers to promote physical activity among adults with disabilities.

## Introduction

In 2008, the US Department of Health and Human Services released *Physical Activity Guidelines for Americans* (*Guidelines*) (1). To obtain substantial health benefits, the *Guidelines* recommend that adults get at least 150 minutes per week of moderate-intensity or 75 minutes per week of vigorous-intensity aerobic physical activity, or an equivalent combination (1). The benefits of physical activity are well known and include a lowered risk of certain chronic conditions, including heart disease, stroke, diabetes, and some cancers (2). Despite these benefits, only 50% of US adults met the aerobic physical activity guideline in 2014 (3).

More than 53 million adults in the United States have disabilities (4). Numerous health-related disparities exist between adults with disabilities and adults without disabilities (5–10), and adults with disabilities are more likely to be physically inactive (5,11,12). However, adults with disabilities should, as they are able, meet the same aerobic physical activity guideline as recommended for all adults (1). When they are not able to achieve this amount, they should avoid inactivity and engage in regular physical activity according to their abilities (1). The *Guidelines* also recommend that adults with disabilities talk to their health care providers about the amounts and types of physical activity appropriate for them (1).

Health care providers are well positioned to recommend physical activity to their patients with disabilities (13,14). Adults with disabilities are more likely than those without disabilities to see a health care provider and have a usual source of care (15,16). A Centers for Disease Control and Prevention (CDC) *Vital Signs* report in 2014 found a significant association among adults with disabilities between receiving a physical activity recommendation from a health care provider and being physically active (12). The report also found that only 4 in 10 adults with disabilities who visited a health care provider in the previous year reported receiving a physical activity recommendation from a health care provider; this percentage was slightly lower among adults without disabilities (12).

Health care provider–reported barriers to physical activity counseling for the general patient population include lack of time, lack of payment, and a perception the patient may not be receptive (17). To our knowledge, reasons health care providers may or may not recommend physical activity to their adult patients with disabilities have not been studied. Therefore, as a follow-up to the *Vital Signs* report, CDC fielded a module of questions to assess primary care providers’ practice of recommending physical activity to adult patients with disabilities. The objective of this study was threefold: 1) to understand perceived level of preparedness among primary care providers in recommending physical activity to their adult patients with disabilities, 2) to describe their characteristics and reported barriers to and knowledge-related factors of recommending physical activity to adults with disabilities; and 3) to determine factors associated with frequency of recommending physical activity to their adult patients with disabilities.

## Methods

Data were from the 2014 DocStyles survey, a web-based survey of primary care physicians and other health professionals conducted annually by Porter Novelli Public Services (18). Porter Novelli commissioned WorldOne (a market research company now merged with SERMO [19]) to draw a randomly selected sample from their Global Medical Panel, which includes more than 270,000 physicians and 1 million medical professionals in the United States. A total of 2,512 health professionals were invited to participate in the survey, with quotas set to reach 1,000 family/general practitioners and internists, 250 pediatricians, 250 obstetricians/gynecologists and 250 nurse practitioners. Screening questions limited the survey to health professionals who practice in the United States, actively see patients, work in an individual or group practice or hospital, and have been in practice for at least 3 years. Of those invited, 132 did not complete the entire survey; 161 were excluded for not meeting the screening criteria; 26 were terminated because of filled quotas, and 433 did not respond or tried to respond after the survey closed, resulting in 1,760 completed surveys and a response rate of 77.5%; family/general practitioners and internists had a response rate of 81.2% and nurse practitioners had a response rate of 74.1% (20). An honorarium of $39 to $73, based on the number of questions posed to each specialty, was paid for survey completion.

Ninety-one questions were asked in 2014, including 8 questions related to physical activity and disability. We further limited our analyses to 1,258 primary care providers who primarily see adult patients (ie, family/general practitioners, internists, and nurse practitioners). We then excluded 49 respondents who reported that they do not see patients with disabilities, resulting in a final sample of 1,209. No individual identifiers were included in the database provided to CDC; institutional review board approval was not necessary.

### Measures

Data on the following demographic and practice characteristics of the respondents included sex (male, female), age group (25–35 y, 36–45 y, 46–55 y, ≥56 y), race/ethnicity (non-Hispanic white, non-Hispanic black, non-Hispanic Asian, Hispanic, non-Hispanic other), financial situation of most of their patients (<lower middle class, lower middle class or middle class, >middle class), type of provider (family/general practitioner, internist, nurse practitioner), main work setting (individual outpatient, group outpatient, inpatient), whether or not they had privileges in a teaching hospital (yes, no), years in practice (3–8 y, 9–14 y, 15–22 y, 23–50 y), average number of adults with disabilities seen per week (<1, 1–5, 6–10, ≥11), and the type of disability most frequently seen in their practice (mobility limitation, emotional or mental health disorder, cognitive limitation, hearing limitation, vision limitation, or none of these).

To determine level of preparedness, we used responses to the statement “I feel prepared to recommend physical activity to my adult patients with disabilities.” Respondents were categorized into 3 groups: 1) strongly agreed, 2) somewhat agreed, and 3) neither agreed nor disagreed (neutral), somewhat disagreed, or strongly disagreed. Because we were interested in primary care providers who agreed they felt prepared and the number of respondents who were neutral or disagreed was small, we combined these responses into a single category.

Frequency of recommending physical activity to adult patients with disabilities was categorized into the following groups: 1) most visits (at every visit or most visits), 2) patient-focused reasons (if the patient brings it up, if the patient is at risk, or it varies/depends on patient needs), 3) other (annual wellness visit, only if time, other), and 4) never. Knowledge-related factors in discussing physical activity were 1) knowledge of the current guideline on aerobic activity (selecting 150 minutes of moderate-intensity aerobic physical activity per week) 2) that the guideline applies to adults with disabilities (yes, no, don’t know), and 3) awareness of community physical activity resources for adults with disabilities. For awareness, respondents were asked to indicate their level of agreement with the following statement: “I am aware of community physical activity resources or programs available for adults with disabilities.” Awareness was categorized as 1) strongly agree, 2) somewhat agree, or 3) neither agreed nor disagreed (neutral), or 4) somewhat disagreed or strongly disagreed. Reported barriers to discussing and recommending physical activity to adult patients with disabilities were categorized as 1) patient-related (lack of patient interest, lack of patient ability to participate, patients have other immediate health issues), 2) provider-related (lack of knowledge of physical activity options for people with disabilities, discomfort in speaking to a patient with a disability about physical activity), 3) system-related (lack of support from practice or health organization, lack of reimbursement, or lack of time), 4) other barrier, and 5) no major barrier. Respondents were asked to choose only one barrier.

### Statistical analysis

We estimated percentages and 95% confidence intervals (CIs) of demographic and practice characteristics of the respondents, both overall and by primary care providers’ level of preparedness to recommend physical activity. We estimated percentages and CIs of physical activity recommendation frequency and knowledge-related factors and barriers to physical activity recommendations by level of preparedness. We tested trends in the percentages of variables across levels of reported preparedness by using Cochran–Armitage tests, Kruskal–Wallis tests, and Spearman correlations, depending on the number of variable levels and whether the variable was ordinal or nominal. We used multivariate log-binomial regression to determine adjusted prevalence ratios (PRs) and 95% CIs of recommending physical activity to adult patients with disabilities at most visits by selected primary care provider characteristics and level of preparedness. We excluded patient characteristics and certain primary care provider characteristics from the model (ie, privileges at a teaching hospital and years of experience, because they were correlated with work setting and age, respectively). In addition, knowledge-related factors and barriers associated with level of preparedness were subsequently excluded from this model. All statistical analyses were performed using SAS version 9.3 (SAS Institute Inc).

## Results

Most respondents were family/general practitioners (44.1%) or internists (37.1%) (Table 1). Most respondents were male (61.5%), non-Hispanic white (62.5%), and in a group outpatient practice (67.2%); 48.1% had privileges at a teaching hospital. Most reported being in practice less than 15 years (28.3%, 3–8 y; 26.6%, 9–14 y). Most saw one or more patients with disabilities per week (16.3% reporting <1 per week), and mobility limitation was the most frequently seen disability (54.6%), followed by emotional or mental health disorder (21.3%).

**Table 1:** Characteristics of Primary Care Providers (N = 1,209), Overall and by Level of Preparedness for Discussing Physical Activity With Their Adult Patients With Disabilities, 2014 DocStyles

Characteristic	Overall	Prepared to Discuss Physical Activity^a^, % (95% Confidence Intervals)			*P* Value^b^
		Strongly Agree	Somewhat Agree	Neutral or Disagree	
**Overall**	100 (0)	36.3 (33.6–39.0)	43.3 (40.6–46.1)	20.4 (18.1–22.6)	NA
**Type of provider**					
Family/general practitioner	44.1 (41.3–46.9)	44.4 (39.8–49.1)	43.9 (39.6–48.1)	43.9 (37.7–50.1)	.79
Internist	37.1 (34.3–39.8)	35.3 (30.8–39.8)	38.7 (34.6–42.9)	36.6 (30.6–42.6)	
Nurse practitioner	18.9 (16.7–21.2)	20.3 (16.5–24.0)	17.4 (14.1–20.6)	19.5 (14.6–24.5)	
**Sex**					
Male	61.5 (58.8–64.3)	62.0 (57.4–66.5)	62.4 (58.3–66.6)	58.9 (52.8–65.1)	.52
Female	38.5 (35.7–41.2)	38.0 (33.5–42.6)	37.6 (33.5–41.7)	41.1 (34.9–47.2)	
**Age group, y**					
25–35	18.0 (15.9–20.2)	17.5 (14.0–21.1)	16.2 (13.1–19.4)	22.8 (17.5–28.0)	.001
36–45	35.4 (32.7–38.1)	29.6 (25.3–33.9)	39.5 (35.3–43.7)	37.0 (31.0–43.0)	
46–55	25.4 (22.9–27.9)	28.5 (24.3–32.7)	23.1 (19.5–26.7)	24.8 (19.4–30.2)	
≥56	21.2 (18.9–23.5)	24.4 (20.4–28.4)	21.2 (17.7–24.7)	15.5 (10.9–20.0)	
**Race/ethnicity**					
Non-Hispanic white	62.5 (59.7–65.2)	65.4 (60.9–69.8)	60.9 (56.7–65.1)	60.6 (54.5–66.7)	.20
Non-Hispanic black	2.3 (1.5–3.2)	3.2 (1.6–4.8)	1.9 (0.7–3.1)	1.6 (0.1–3.2)	
Non-Hispanic Asian	23.3 (20.9–25.7)	19.6 (15.9–23.3)	25.8 (22.0–29.5)	24.8 (19.4–30.2)	
Hispanic	5.0 (3.7–6.2)	5.0 (3.0–7.1)	4.6 (2.8–6.4)	5.7 (2.8–8.6)	
Non-Hispanic other^c^	7.0 (5.5–8.4)	6.8 (4.5–9.2)	6.9 (4.7–9.0)	7.3 (4.1–10.6)	
**Financial situation of most patients**					
<Lower middle class	18.8 (16.6–21.0)	18.2 (14.6–21.8)	19.1 (15.7–22.5)	19.1 (14.2–24.0)	.82
Lower middle class or middle class	39.5 (36.7–42.2)	41.5 (36.9–46.1)	37.8 (33.6–41.9)	39.4 (33.3–45.5)	
>Middle class	41.8 (39.0–44.6)	40.3 (35.7–44.9)	43.1 (38.9–47.4)	41.5 (35.3–47.6)	
**Main work setting**					
Individual outpatient	19.0 (16.8–21.2)	20.5 (16.7–24.3)	17.9 (14.7–21.2)	18.7 (13.8–23.6)	.11
Group outpatient	67.2 (64.5–69.8)	67.7 (63.3–72.0)	68.5 (64.5–72.5)	63.4 (57.4–69.4)	
Inpatient	13.8 (11.9–15.8)	11.9 (8.8–14.9)	13.6 (10.6–16.5)	17.9 (13.1–22.7)	
**Privileges at teaching hospital**					
Yes	48.1 (45.3–51.0)	49.2 (44.5–53.9)	47.1 (42.9–51.4)	48.4 (42.1–54.6)	.75
No	51.9 (49.0–54.7)	50.8 (46.1–55.5)	52.9 (48.6–57.1)	51.6 (45.4–57.9)	
**Years in practice**					
3–8	28.3 (25.8–30.8)	27.6 (23.4–31.7)	26.7 (22.9–30.5)	32.9 (27.1–38.8)	.009
9–14	26.6 (24.1–29.0)	22.1 (18.2–26.0)	30.9 (27.0–34.9)	25.2 (19.8–30.6)	
15–22	23.1 (20.7–25.5)	24.8 (20.8–28.9)	19.9 (16.4–23.3)	26.8 (21.3–32.4)	
23–50	22.1 (19.8–24.4)	25.5 (21.4–29.6)	22.5 (18.9–26.1)	15.0 (10.6–19.5)	
**Average no. of patients with disabilities seen weekly**					
<1	16.3 (14.2–18.4)	12.8 (9.6–15.9)	16.6 (13.4–19.8)	22.0 (16.8–27.1)	<.001
1–5	38.2 (35.5–41.0)	34.2 (29.7–38.6)	40.1 (35.9–44.3)	41.5 (35.3–47.6)	
6–10	22.2 (19.8–24.5)	24.6 (20.6–28.6)	21.0 (17.5–24.5)	20.3 (15.3–25.4)	
≥11	23.3 (20.9–25.7)	28.5 (24.3–32.7)	22.3 (18.8–25.9)	16.3 (11.7–20.9)	
**Type of disability most frequently seen**					
Mobility limitation	54.6 (51.8–57.4)	58.8 (54.2–63.4)	51.9 (47.6–56.2)	52.9 (46.6–59.1)	.13
Emotional or mental health disorder	21.3 (19.0–23.6)	20.7 (16.9–24.5)	22.3 (18.8–25.9)	19.9 (14.9–24.9)	
Cognitive limitation	10.8 (9.1–12.6)	10.3 (7.4–13.1)	10.1 (7.5–12.7)	13.4 (9.2–17.7)	
Hearing limitation	7.8 (6.3–9.3)	5.9 (3.7–8.1)	9.4 (6.9–11.8)	7.7 (4.4–11.1)	
Vision limitation	4.7 (3.5–5.9)	4.3 (2.4–6.2)	5.0 (3.1–6.8)	4.9 (2.2–7.6)	
None of these	0.8 (0.3–1.3)	0 (0)	1.3 (0.4–2.3)	1.2 (0.0–2.6)	

a Categories are based on responses to following statement: “I feel prepared to recommend physical activity to my adult patients with disabilities.” Respondents were categorized into 3 groups: 1) strongly agreed, 2) somewhat agreed, and 3) neither agreed nor disagreed (neutral), somewhat disagreed, or strongly disagreed. Because we were interested in primary care providers who agreed they felt prepared and because the number of respondents who were neutral or disagreed was small, we combined these responses into a single category.

b Based on the appropriate test for trend on the distribution of the characteristic across the levels preparedness, including Cochran–Armitage test, Kruskal–Wallis test, and Spearman correlation.

c Other includes Native Hawaiian or Other Pacific Islander, American Indian or Alaska Native, ≥ 2 races, and other race.

Overall, most respondents reported they strongly (36.3%) or somewhat (43.3%) agreed they felt prepared to recommend physical activity to their patients with disabilities (Table 1). We found a significant trend between level of preparedness and both primary care provider age and years of practice. Nearly one-quarter (24.4%) of primary care providers who strongly agreed were aged 56 years or older, while 21.2% of those who somewhat agreed and 15.5% who were neutral or disagreed were in that age group. In addition, we found a significant trend between preparedness and number of patients with disabilities seen per week; 28.5% of those who strongly agreed that they felt prepared saw 11 or more patients with disabilities per week compared with 16.3% who were neutral or disagreed. We found no other significant trends in primary care provider characteristics by level of preparedness.

About half (50.6%) of primary care providers reported recommending physical activity to patients with disabilities at most clinic visits; we found a significant trend in frequency of recommending physical activity and preparedness level. Nearly two-thirds (62.0%) of primary care providers who strongly agreed they felt prepared, 48.5% who somewhat agreed, and 35.0% who were neutral or disagreed reported recommending physical activity at most visits (Table 2). Conversely, although 35.7% of primary care providers overall recommended physical activity based on patient-focused reasons, 29.2% who strongly agreed, 36.5% who somewhat agreed, and 45.9% who were neutral or disagreed reported this recommendation frequency. While only 17.3% of primary care providers overall strongly agreed they were aware of community physical activity resources, 34.2% of primary care providers who strongly agreed they felt prepared to discuss physical activity also strongly agreed they were aware of community physical activity resources. We found a significant trend between preparedness level and awareness of community resources. Although about half (53.7%) of primary care providers knew the guideline on aerobic activity applied to adults with disabilities, the percentage was highest among those who strongly agreed they felt prepared (62.0%) and lowest (40.2%) among those who were neutral or disagreed.

**Table 2:** Percentage of Primary Care Providers (N = 1,209) Recommending Physical Activity and Knowledge-Related Factors and Barriers to Recommending Physical Activity, by Level of Preparedness for Discussing Physical Activity With Their Adult Patients With Disabilities, 2014 DocStyles

Characteristic	Overall	Prepared to Discuss Physical Activity,^a^ % (95% Confidence Intervals)			*P* Value^b^
		Strongly Agree	Somewhat Agree	Neutral or Disagree	
**How often recommend physical activity**					
Every visit or most clinic visits	50.6 (47.8–53.4)	62.0 (57.4–66.5)	48.5 (44.2–52.8)	35.0 (29.0–40.9)	<.001
Patient-focused reasons^c^	35.7 (33.0–38.4)	29.2 (24.9–33.4)	36.5 (32.3–40.6)	45.9 (39.7–52.2)	
Other (annual wellness visit, only if time, other)	11.8 (9.9–13.6)	8.7 (6.0–11.3)	13.7 (10.8–16.7)	13.0 (8.8–17.2)	
Never	1.9 (1.1–2.7)	0.2 (0.0–0.7)	1.3 (0.4–2.3)	6.1 (3.1–9.1)	
**Knowledge-Related Factors**					
**Awareness of community physical activity resources^d^**					
Strongly agree	17.3 (15.2–19.4)	34.2 (29.7–38.6)	10.1 (7.5–12.7)	2.4 (0.5–4.4)	<.001
Somewhat agree	44.8 (41.9–47.6)	42.4 (37.8–47.0)	54.0 (49.7–58.3)	29.3 (23.6–35.0)	
Neither	20.4 (18.1–22.6)	12.5 (9.4–15.6)	21.8 (18.2–25.3)	31.3 (25.5–37.1)	
Strongly/somewhat disagree	17.6 (15.5–19.8)	10.9 (8.0–13.9)	14.1 (11.1–17.1)	37.0 (31.0–43.0)	
**Knowledge of the guideline on aerobic activity^e^**					
Provider’s knowledge is accurate	18.5 (16.3–20.7)	17.8 (14.2–21.3)	17.8 (14.5–21.0)	21.5 (16.4–26.7)	.24
**The guideline on aerobic activity applies to adults with disabilities**					
Yes	53.7 (50.9–56.5)	62.0 (57.4–66.5)	53.1 (48.8–57.3)	40.2 (34.1–46.4)	<.001
No	8.9 (7.3–10.5)	9.8 (7.0–12.6)	8.0 (5.7–10.3)	8.9 (5.4–12.5)	
Don’t know	37.5 (34.7–40.2)	28.3 (24.0–32.5)	38.9 (34.8–43.1)	50.8 (44.6–57.1)	
**Barriers**					
**Patient-related**	49.5 (46.6–52.3)	54.0 (49.3–58.7)	49.1 (44.8–53.3)	42.3 (36.1–48.5)	.02^f^
Lack of patient interest in physical activity	25.9 (23.4–28.4)	31.0 (26.7–35.3)	24.6 (20.9–28.3)	19.5 (14.6–24.5)	
Lack of patient ability to participate in physical activity	9.8 (8.2–11.5)	9.1 (6.4–11.8)	11.6 (8.9–14.4)	7.3 (4.1–10.6)	
Patients have other immediate health issues	13.7 (11.8–15.7)	13.9 (10.7–17.1)	12.8 (9.9–15.7)	15.5 (10.9–20.0)	
**Provider-related**	15.9 (13.8–17.9)	13.2 (10.0–16.4)	15.8 (12.7–19.0)	20.7 (15.7–25.8)	
Lack of knowledge about physical activity options for people with disabilities	13.1 (11.2–15.0)	10.0 (7.2–12.8)	13.2 (10.3–16.1)	18.3 (13.5–23.1)	
Discomfort in speaking to patient with disability about physical activity	2.8 (1.9–3.7)	3.2 (1.6–4.8)	2.7 (1.3–4.1)	2.4 (0.5–4.4)	
**System-related**	26.5 (24.0–29.0)	23.2 (19.3–27.2)	28.6 (24.8–32.5)	27.6 (22.1–33.2)	
Lack of support from practice or health organization	4.2 (3.1–5.4)	4.6 (2.6–6.5)	3.4 (1.9–5.0)	5.3 (2.5–8.1)	
Lack of reimbursement	6.3 (4.9–7.7)	6.6 (4.3–9.4)	5.7 (3.7–7.7)	6.9 (3.7–10.1)	
Lack of time	16.0 (13.9–18.0)	12.1 (9.0–15.1)	19.5 (16.1–22.9)	15.5 (10.9–20.0)	
**Other barrier**	1.4 (0.7–2.1)	1.6 (0.4–2.8)	1.3 (0.4–2.3)	1.2 (0.0–2.6)	
**No major barriers**	6.8 (5.4–8.2)	8.0 (5.4–10.5)	5.2 (3.3–7.1)	8.1 (4.7–11.6)	

a Categories are based on responses to following statement: “I feel prepared to recommend physical activity to my adult patients with disabilities.” Respondents were categorized into 3 groups: 1) strongly agreed, 2) somewhat agreed, and 3) neither agreed nor disagreed (neutral), somewhat disagreed, or strongly disagreed. Because we were interested in primary care providers who agreed they felt prepared and because the number of respondents who were neutral or disagreed was small, we combined these responses into a single category.

b Based on the appropriate test for trend on the distribution of the characteristic across the levels of preparedness, including Cochran–Armitage test, Kruskal–Wallis test, and Spearman correlation.

c If the patient brings it up or if the patient is at risk, or it varies or depends on patient needs.

d Based on responses to the following statement: “I am aware of community physical activity resources or programs available for adults with disabilities.”

e At least 150 minutes per week of moderate-intensity or 75 minutes per week of vigorous-intensity aerobic physical activity, or an equivalent combination (1).

f Test for trend across the 5 overall categories using a Kruskal–Wallis test.

The most common barriers reported to discussing and recommending physical activity to adult patients with disabilities were patient related (Table 2). These barriers were most frequently reported by those who strongly agreed they felt prepared (54.0%) and decreased as level of preparedness decreased. Provider-related barriers were reported by 15.9% of respondents; 20.7% of respondents who were neutral or disagreed they felt prepared reported this as their primary barrier. System-related barriers were reported by 26.5% of primary care providers; the lowest percentage was among those who strongly agreed they felt prepared (23.2%).

Level of preparedness was significantly associated with recommending physical activity at most visits, controlling for primary care provider characteristics (Table 3). Those who strongly agreed (adjusted PR, 1.74; 95% CI, 1.44–2.09) or somewhat agreed (adjusted PR, 1.36; 95% CI, 1.22–1.65) they felt prepared were more likely to recommend physical activity at most visits compared with those who were neutral or disagreed. Primary care providers seeing 6 to 10 patients with disabilities (adjusted PR, 1.32; 95% CI, 1.08–1.60) or 11 or more patients with disabilities per week (adjusted PR, 1.32; 95% CI, 1.09–1.60) were also more likely to recommend physical activity at most visits compared with those seeing less than 1 patient with disabilities per week.

**Table 3:** Association of Characteristics^a^ of Primary Care Providers (N = 1,209) With Recommending Physical Activity at Every or Most Visits to Adult Patients With Disabilities, From Multivariate Log-Binomial Regression, 2014 DocStyles

Characteristic	Adjusted Prevalence Ratio (95% Confidence Interval)	*P* Value
**Prepared to discuss physical activity^b^**		
Strongly agree	1.74 (1.44–2.09)	<.001
Somewhat agree	1.36 (1.22–1.65)	.002
Neutral/disagree	1 [Reference]	
**Type of provider**		
Family/general practitioner	0.94 (0.80–1.10)	.44
Internist	0.98 (0.82–1.17)	.81
Nurse practitioner	1 [Reference]	
**Sex**		
Male	1.01 (0.89–1.14)	.89
Female	1 [Reference]	
**Age group, y**		
25–35	1.12 (0.94–1.32)	.20
36–45	0.96 (0.82–1.12)	.61
46–55	0.97 (0.83–1.13)	.72
≥56 years	1 [Reference]	
**Race/ethnicity**		
Non-Hispanic white	0.92 (0.75–1.14)	.46
Non-Hispanic black	0.85 (0.55–1.32)	.47
Non-Hispanic Asian	1.11 (0.90–1.38)	.32
Hispanic	0.89 (0.65–1.23)	.48
Non-Hispanic other^c^	1 [Reference]	
**Main work setting**		
Individual outpatient	1.07 (0.87–1.31)	.53
Group outpatient	1.00 (0.84–1.18)	.98
Inpatient	1 [Reference]	
**Average no. of patients with disabilities seen weekly**		
<1	1 [Reference]	
1–5	1.14 (0.94–1.38)	.18
6–10	1.32 (1.08–1.60)	.006
≥11	1.32 (1.09–1.60)	.005

a All variables included in the model are reported in the table.

b Categories are based on responses to following statement: “I feel prepared to recommend physical activity to my adult patients with disabilities.” Respondents were categorized into 3 groups: 1) strongly agreed, 2) somewhat agreed, and 3) neither agreed nor disagreed (neutral), somewhat disagreed, or strongly disagreed. Because we were interested in primary care providers who agreed they felt prepared and because the number of respondents who were neutral or disagreed was small, we combined these responses into a single category.

c Other includes Native Hawaiian or Other Pacific Islander, American Indian or Alaska Native, ≥ 2 races, and other race.

## Discussion

We found that just over one-third of primary care providers strongly agreed they felt prepared to discuss physical activity with their adult patients with disabilities, whereas about 20% were neutral or disagreed. Primary care provider age, number of patients with disabilities seen per week, and awareness of community physical activity resources were significantly associated with level of preparedness. Additionally, those who strongly agreed they felt prepared had nearly a 75% higher prevalence of recommending physical activity to their patients with disabilities at most visits compared with those who were neutral or disagreed. Identifying ways to help primary care providers feel more prepared to recommend and discuss physical activity with their adult patients with disabilities may help ensure that primary care providers can be a resource to promote physical activity among this population.

Seeing more patients with disabilities per week was significantly associated both with recommending physical activity at most visits and with how prepared the respondent felt. This finding suggests that experience interacting with this population may affect how prepared primary care providers feel and how often they discuss physical activity with their patients with disabilities. Increasing exposure to people with disabilities in medical school curricula and developing disability-related competencies for health professionals are proposed as ways to enhance providers’ ability to address the needs of this population (21,22).

We found a positive association between preparedness and awareness of community physical activity resources; more than one-third of primary care providers who strongly agreed they felt prepared also strongly agreed they were aware of such resources. Although only 10% of primary care providers who felt prepared reported lack of knowledge about resources as their primary barrier to recommending physical activity, this percentage nearly doubled among primary care providers who were neutral or disagreed. Awareness of community resources may be particularly important, because receiving information about such resources in conjunction with advice to engage in activity can be more helpful to patients than physical activity–related counseling alone (21). Increasing primary care provider awareness of national, state, and local resources for people with disabilities (eg, YMCAs, local chapters of Special Olympics, Arthritis Foundation programs, accessible parks and recreations centers) may assist primary care providers in feeling more prepared to address and discuss physical activity options with their patients with disabilities. Additionally, public health programs and other organizations have resources to help primary care providers prepare to discuss physical activity with their patients with disabilities, including the National Center on Health, Physical Activity and Disability’s Physician Toolkit (23), Exercise is Medicine (24), and CDC’s Disability and Health Physical Activity website (25).

The most frequently reported category of barriers was patient-related. One such barrier was the perception that patients with disabilities have other immediate health needs. Although intensive behavioral counseling on physical activity is not explicitly recommended for the general population (26), it is for those at high risk for certain chronic conditions, such as cardiovascular disease (27,28). Because many chronic conditions, including cardiovascular disease, are more prevalent among adults with disabilities than among adults without disabilities (5,12), primary care providers might consider focusing on the chronic condition risk status of their patients with disabilities in considering physical activity counseling for this population.

Other patient-related barriers were perceptions that patients lack interest or are unable to participate in physical activity. One possible solution may be to weave a recommendation for physical activity into discussions of other related health issues, such as falls, balance, and endurance, rather than addressing physical activity separately (29). Merging these discussions may also assist primary care providers who cite lack of time as their primary barrier (16% of primary care providers in our study). In addition, some patients with disabilities may be unable to participate in the amounts of physical activity recommended by the *Guidelines.* Most (81.5%) primary care providers in our study were not able to identify the current aerobic physical activity guideline, and the survey did not ask primary care providers how much physical activity they are recommending to their patients with disabilities. The *Guidelines* state that adults with disabilities should participate in physical activity as they are able and avoid inactivity (1). Primary care providers can help patients with disabilities identify activities consistent with their abilities and encourage them to participate in shorter bouts of activity to increase their activity level. Furthermore, because socioeconomic disparities (ie, lower income and levels of education) (5) exist between adults with disabilities and adults without disabilities, adults with disabilities may face additional barriers, such as costs and transportation (30). Recommendation of a home-based program (30) or a low-cost accessible community program (25) may support patient participation.

This study has several limitations. First, our results may not be representative of all US health professionals, and they may reflect only the perspectives of primary care providers. However, the distribution of age, sex, years of practice, and regional locations of the 2014 DocStyles sample was similar to that of the 2014 American Medical Association master file (20). Second, our results may be subject to nonresponse bias; primary care providers who participated may have answered survey questions differently than primary care providers who did not participate. Third, responses are self-reported and may be subject to social desirability bias, thus, certain items may have been overreported or underreported. Fourth, all responses are from the perspective of the primary care provider and may not represent their patients’ perspectives. Finally, DocStyles questions are not evaluated for reliability or validity. However, DocStyles is developed with input from clinical medicine and public health experts and provides a unique perspective of US health professionals.

Health disparities among people with disabilities are well documented (5–7,9,10), including the greater likelihood of being physically inactive compared with people without disabilities (11,12). The current *Guidelines* recommend that adults with disabilities consult with their health care providers about how much and what type of physical activity is best for them (1), and a provider recommendation is associated with physical activity participation among this group (12). We found that those who feel prepared to discuss physical activity with their adult patients with disabilities are more likely to recommend it to them regularly. Increasing primary care provider knowledge and use of existing public health tools may facilitate provider recommendation of physical activity to adults with disabilities. Further understanding of barriers primary care providers face can aid in developing additional targeted public health resources.

## References

[1] US Department of Health and Human Services. Physical activity guidelines for Americans. Washington (DC): Office of Disease Prevention and Health Promotion; 2008. https://health.gov/paguidelines/pdf/paguide.pdf. Accessed June 26, 2017.

[2] Physical Activity Guidelines Advisory Committee. Physical Activity Guidelines Advisory Committee Report, 2008. Washington (DC): US Department of Health and Human Services; 2008. https://health.gov/paguidelines/report/pdf/CommitteeReport.pdf. Accessed June 26, 2017. 10.1111/j.1753-4887.2008.00136.x 19178654

[3] National Center for Health Statistics. Health, United States, 2015: with special feature on racial and ethnic health disparities. Hyattsville (MD): National Center for Health Statistics; 2016. https://www.cdc.gov/nchs/data/hus/hus15.pdf#summary. Accessed June 26, 2017. 27308685

[4] Courtney-Long EA , Carroll DD , Zhang QC , Stevens AC , Griffin-Blake S , Armour BS , Prevalence of disability and disability type among adults — United States, 2013. MMWR Morb Mortal Wkly Rep 2015;64(29):777–83. 10.15585/mmwr.MM6429a2 26225475 PMC4584831

[5] Pharr JR , Bungum T . Health disparities experienced by people with disabilities in the United States: a Behavioral Risk Factor Surveillance System study. Glob J Health Sci 2012;4(6):99–108. 10.5539/gjhs.v4n6p99 23121746 PMC4776960

[6] Courtney-Long E , Armour B , Frammartino B , Miller J . Factors associated with self-reported mammography use for women with and women without a disability. J Womens Health (Larchmt) 2011;20(9):1279–86. 10.1089/jwh.2010.2609 21732810

[7] Courtney-Long E , Stevens A , Caraballo R , Ramon I , Armour BS . Disparities in current cigarette smoking prevalence by type of disability, 2009–2011. Public Health Rep 2014;129(3):252–60. 10.1177/003335491412900307 24791023 PMC3982553

[8] Steele CB , Townsend JS , Courtney-Long EA , Young M . Prevalence of cancer screening among adults with disabilities, United States, 2013. Prev Chronic Dis 2017;14:E09. 10.5888/pcd14.160312 28125399 PMC5268742

[9] Stevens A , Courtney-Long E , Gillespie C , Armour BS . Hypertension among US adults by disability status and type, National Health and Nutrition Examination Survey, 2001–2010. Prev Chronic Dis 2014;11:E139. 10.5888/pcd11.140162 25121351 PMC4133509

[10] Armour BS , Courtney-Long EA , Campbell VA , Wethington HR . Disability prevalence among healthy weight, overweight, and obese adults. Obesity (Silver Spring) 2013;21(4):852–5. 10.1002/oby.20312 23712989

[11] Centers for Disease Control and Prevention. Physical activity among adults with a disability — United States, 2005. MMWR Morb Mortal Wkly Rep 2007;56(39):1021–4. 17914329

[12] Carroll DD , Courtney-Long EA , Stevens AC , Sloan ML , Lullo C , Visser SN , Vital signs: disability and physical activity — United States, 2009–2012. MMWR Morb Mortal Wkly Rep 2014;63(18):407–13. 24807240 PMC5779402

[13] Patrick K , Pratt M , Sallis RE . The healthcare sector’s role in the U.S. national physical activity plan. J Phys Act Health 2009;6(S2):S211–9. 10.1123/jpah.6.s2.s211 28872433

[14] AuYoung M , Linke SE , Pagoto S , Buman MP , Craft LL , Richardson CR , Integrating physical activity in primary care practice. Am J Med 2016;129(10):1022–9. 10.1016/j.amjmed.2016.02.008 26953063

[15] Iezzoni LI , Frakt AB , Pizer SD . Uninsured persons with disability confront substantial barriers to health care services. Disabil Health J 2011;4(4):238–44. 10.1016/j.dhjo.2011.06.001 22014671

[16] Sommers AS . Access to health insurance, barriers to care, and service use among adults with disabilities. Inquiry 2006–2007;43(4):393–405. 10.5034/inquiryjrnl_43.4.393 17354373

[17] Eakin EG , Smith BJ , Bauman AE . Evaluating the population health impact of physical activity interventions in primary care — are we asking the right questions? J Phys Act Health 2005;2(2):197–215. 10.1123/jpah.2.2.197

[18] Porter Novelli Public Services. DocStyles 2014 [raw data]. Washington (DC): Deanne Weber; 2014.

[19] SERMO. Research panel. http://www.sermo.com/business-solutions/introduction#our-research. Accessed October 5, 2017.

[20] Porter Novelli. DocStyles 2014 methods. Washington (DC): Deanne Weber; 2014.

[21] Kirschner KL , Curry RH . Educating health care professionals to care for patients with disabilities. JAMA 2009;302(12):1334–5. 10.1001/jama.2009.1398 19773571

[22] Symons AB , McGuigan D , Akl EA . A curriculum to teach medical students to care for people with disabilities: development and initial implementation. BMC Med Educ 2009;9(1):78. 10.1186/1472-6920-9-78 20042110 PMC2809044

[23] Physician’s toolkit from The National Center on Health Physical Activity and Disability. Birmingham (AL): The National Center on Health Physical Activity and Disability; 2017. http://www.nchpad.org/1195/5822/Physician~s~Toolkit. Accessed April 13, 2017.

[24] Exercise is medicine. Indianapolis (IN): American College of Sports Medicine; 2017. http://www.exerciseismedicine.org/. Accessed April 14, 2017.

[25] Increasing physical activity among adults with disabilities. Atlanta (GA): US Department of Health and Human Services, Centers for Disease Control and Prevention, National Center on Birth Defects and Developmental Disabilities, Division of Human Development and Disability; 2016. https://www.cdc.gov/ncbddd/disabilityandhealth/pa.html. Accessed April 14, 2017.

[26] Grossman DC , Bibbins-Domingo K , Curry SJ , Barry MJ , Davidson KW , Doubeni CA , Behavioral counseling to promote a healthful diet and physical activity for cardiovascular disease prevention in adults without cardiovascular risk factors: U.S. Preventive Services Task Force recommendation statement. JAMA 2017;318(2):167–74. 10.1001/jama.2017.7171 28697260

[27] LeFevre ML , U.S. Preventive Services Task Force. Behavioral counseling to promote a healthful diet and physical activity for cardiovascular disease prevention in adults with cardiovascular risk factors: U.S. Preventive Services Task Force Recommendation Statement. Ann Intern Med 2014;161(8):587–93. 10.7326/M14-1796 25155419

[28] Omura JD , Carlson SA , Paul P , Watson KB , Loustalot F , Foltz JL , Adults eligible for cardiovascular disease prevention counseling and participation in aerobic physical activity — United States, 2013. MMWR Morb Mortal Wkly Rep 2015;64(37):1047–51. 10.15585/mmwr.mm6437a4 26401758

[29] Bardach SH , Schoenberg NE . The content of diet and physical activity consultations with older adults in primary care. Patient Educ Couns 2014;95(3):319–24. 10.1016/j.pec.2014.03.020 24736190 PMC4058830

[30] Rimmer JH , Rubin SS , Braddock D . Barriers to exercise in African American women with physical disabilities. Arch Phys Med Rehabil 2000;81(2):182–8. 10.1016/S0003-9993(00)90138-2 10668772

